# MLC positional accuracy evaluation through the Picket Fence test on EBT2 films and a 3D volumetric phantom

**DOI:** 10.1120/jacmp.v16i2.5185

**Published:** 2015-03-08

**Authors:** Christos Antypas, Ioannis Floros, Maritina Rouchota, Christina Armpilia, Maria Lyra

**Affiliations:** ^1^ 1^st^ Department of Radiology Medical Physics Unit, Aretaieion Hospital, University of Athens Greece

**Keywords:** Picket Fence, radiochromic film, 3D volumetric phantom, MLC, quality assurance

## Abstract

The accuracy of MLC positions during radiotherapy is important as even small positional deviations can translate into considerable dose delivery errors. This becomes crucial when radiosensitive organs are located near the treated volume and especially during IMRT, where dose gradients are steep. A test commonly conducted to measure the positional accuracy of the MLCs is the Picket Fence test. In this study two alterations of the Picket Fence test were performed and evaluated, the first one using radiochromic EBT2 films and the second one the ${\text{Delta}}^{4\text{PT}}$ diode array phantom and its software. Our results showed that EBT2 films provide a relatively fast, qualitative visual inspection of the significant leaf dispositions. When slight inaccuracies need to be revealed or precise numerical results for each leaf position are needed, ${\text{Delta}}^{4\text{PT}}$ provides the desired accuracy of 1 mm. In treatment modalities where a higher accuracy is required in the delivered dose distribution, such as in IMRT, precise numerical values of the measurements for the MLC positional inspection are required.

PACS number: 87.55.Qr, 87.56.bd, 87.56.Fc, 87.56.nk

## I. INTRODUCTION

In intensity‐modulated radiation therapy (IMRT), the radiation fluence of different beam directions is combined to produce a specific dosimetric distribution around a target volume. The final distribution can result in high local doses and steep dose gradients within the irradiated volume.^(1)^ Furthermore, vital organs need to be taken into account when they are located near the boundaries of a high‐dose irradiated area. For these reasons, the accuracy in the delivered dose is vital in IMRT.

The accuracy of the multileaf collimator (MLC) positions is one of the factors that most greatly influences the resultant precision in dose delivery.^(2)^ Even small systematic MLC positional inaccuracies can translate in determinative dose delivery errors, both to tumor and sensitive anatomic structures.^(3)^ The association between the two inaccuracies has been studied by LoSasso^(4)^ and has shown that for a typical 2 cm MLC gap, often encountered in prostate and head and neck fields, a systematic gap error of 1 mm will produce an average dose delivery error of 5%. As far as random MLC errors are concerned, for errors up to 2 mm the dosimetric effect has been found to be negligible.^(5)^ The goal in every radiation therapy procedure is to be able to deliver the prescribed dose with an overall uncertainty of less than 5%^1^, ^6^ or even better.

A common test conducted to measure the positional accuracy of the MLC is the Picket Fence test. This test provides an assessment of the position of each MLC leaf individually and in relation to the alignments of the other leaves.^(4)^ It also shows the actual irradiated gap width. In the literature there are two proposed methodologies to conduct the test: either by creating a uniform pattern with abutting fields^7^, ^8^ or by using specified intervals to irradiate a series of narrow bands.^9^, ^10^ In the first proposed method, the resulting image is checked for missed or overirradiated spots. In the second one, the width of the narrow bands is measured and checked for discrepancies. In this study the second method was followed.

## II. MATERIALS AND METHODS

### A. The linac and MLC system

The measurements were carried out on a 6 MV Siemens ONCOR linear accelerator (Siemens, Malvern, PA). This system utilizes a double‐focused MLC (OPTIFOCUS; Siemens) designed in 41 leaf pairs, in the X direction, that project to 1 cm width at 100 cm from the source. This provides coverage of a full 40 cm IMRT field length. The maximum leaf movement of a single leaf is 30 cm which includes 10 cm overtravel. The double‐focus leaf design follows the beam divergence in both directions, producing a relatively narrow beam penumbra.

### B. Radiochromic EBT2 films

Radiochromic films present an easier alternative to radiographic films, commonly used for the Picket Fence test.^(4)^ Radiochromic films do not need processing as they are self‐developing and permanent color changes occur without the need for chemical developing. They are nearly tissue‐equivalent, they are not affected by indoor lighting, they present a high spatial resolution, and are water resistant.^11^, ^12^ They present a high uniformity and can respond accurately in a wide dose range, between 1 and 50 Gy.^(13)^ Radiochromic films can also be used for dosimetric controls, as they can reveal a two‐dimensional (2D) optical density fluence map of the irradiated area, easily converted to a 2D dose map.

### C. The ${\text{Delta}}^{4\text{PT}}$ phantom

**Figure 1:**
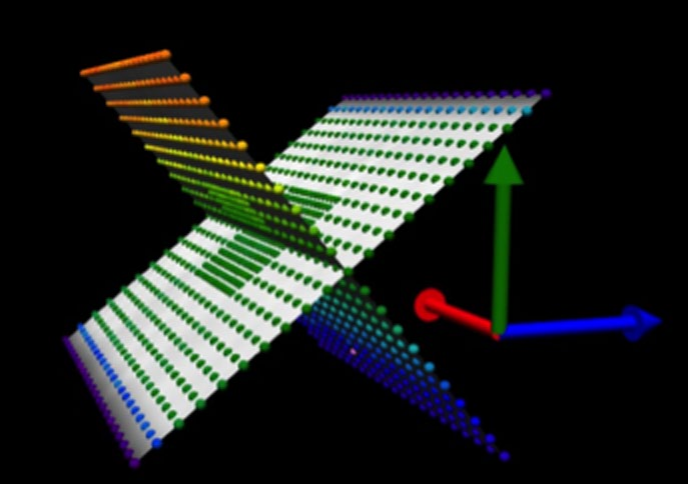
The two detector planes of the ${\text{Delta}}^{4\text{PT}}$ phantom by ScandiDos. Different colors indicate the deviation between planned and delivered dose.^(15)^

The three‐dimensional (3D) volumetric phantom ${\text{Delta}}^{4\text{PT}}$ phantom by ScandiDos (Uppsala, Sweden) was used in this study. The phantom is constructed of PMMA and is designed in two planes (i.e., wings), consisting of a total of 1069 p‐type silicon (Si) detectors (Fig. 1). The distance between the neighboring detectors is of 5 mm in the center of the phantom and of 10 mm in the outer phantom area. The detectors' dose resolution is of 0.01 mGy and the dose response threshold is of 1 mGy.^(14)^

A calibration of the three phantom wings is required at least once a year. Our linac is a one‐energy modality, so calibration was performed for the 6 MV energy. The recommended calibration process by ScandiDos^(15)^ including the wing uniformity response, directional dependence, and absolute dose calibration, was followed.

The ${\text{Delta}}^{4\text{PT}}$ and its software provides the ability to perform a linac QA check for both the beam parameter constancy (profiles and depth doses, trend analysis) and the MLC performance through the Picket Fence (gap width and leaf positions).

### D. EBT2 film calibration

A Gafchromic EBT2 film by International Specialty Products (ISP, Wayne, NJ), sized $25.4\times 20.3\;{\text{cm}}^{2}$, was used.

A calibration of the EBT2 films was first required. Film samples from the same batch were cut ($3\times 20\;{\text{cm}}^{2}$) and placed between sheets of solid water (SP34 QA Phantom, Scanditronix‐Wellhofer, Schuarzenbruck, Germany) with 10 cm of buildup material above and below the film and a source to surface distance (SSD) of 100 cm. The film samples were irradiated perpendicularly to the axis of the beam, with increasing levels of MU. Irradiations were performed with 50−350 MUs (step of 50 MUs), corresponding to doses of 33.5−250 cGy (step of 33.5 cGy) at 10 cm depth. The raw film samples were then scanned with an Epson V750 flatbed scanner (US Epson, Long Beach, CA) in a tiff format, with a resolution of 75 dpi and 24 bits, and measured through the ImageJ analysis software (National Institute of Health, Bethesda, MD), after background was subtracted.^(11)^

The scan resolution of 75 dpi was chosen, as this corresponds to a submillimeter pixel size, adequate for the desired imaging accuracy.

### E. The Picket Fence test with the EBT2 films

The Picket Fence was performed on a radiochromic film, following a motion pattern suggested for films.^(4)^ It consisted of a series of step‐and‐shoot measurements (beam cycled off and on), creating narrow bands at specified intervals. The film was placed on the treatment table at the isocenter level, with a source‐to‐film distance (SFD) of 100 cm and without any additional buildup to create a sharper image. The gantry angle was set to zero degrees and 11 narrow bands were irradiated at specified intervals, separated by 2 cm distance, with nominal gap widths of 3 mm. The irradiation was performed with 250 MUs per field (a total of 2750 MUs) to capacitate a wide range of gray levels, and thus covering a wide range of dose levels.

It has been reported in the literature that radiochromic films, like the EBT2, undergo slight postexposure changes.^(16)^ However, for the dose levels under study, an immediate scanning could have been performed since, the variations in net optical density (OD) between 1 hr and 24 hr after irradiation have been measured to be less than 0.01.^(17)^ However, in order to ensure that the film darkening had stabilized when scanned, the film scanning was performed at 24 hr after the irradiation. The film was scanned under the same conditions as during calibration, without any additional color correction, to capacitate all the advantages of multichannel dosimetry. This allows for the dose‐dependent and dose‐independent parts of the scanned signal to be separated and for the entire available sensitivity range of the film to be empowered in the same procedure.^(18)^

After scanning, the raw images of the irradiated film were imported to the ImageJ (1.38x) Wayne Rasband National Institute of Health USA) analysis software for further processing.

### F. The Picket Fence test as applied on the ${\text{Delta}}^{4\text{PT}}$ phantom

An alteration of the picket fence test was applied on the ${\text{Delta}}^{4\text{PT}}$ phantom.

Before each set of measurements is performed two $10\times 10\;{\text{cm}}^{2}$ fields are delivered, with 100 cGy each, at gantry angles of 0° and 90°. These measurements are then used for output and setup correction purposes.

The phantom was placed isocentrically on the treatment table, at an SAD of 100 cm. The gantry angle was set at 320°, perpendicular to the detectors' plane and each irradiation was performed with 5 MUs.

**Figure 2:**
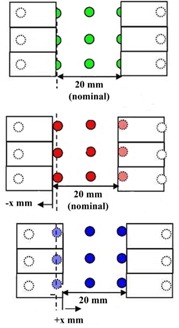
The motion pattern of the picket fence test in the ${\text{Delta}}^{4\text{PT}}$ software. At each major stop of the leaves three irradiations are being delivered: the first with the detector row in the middle of the 2 cm gap with no offset, the second with an offset to the one direction, and the third with the offset to the other direction.^(15)^

The MLC leaves in this method follow a slightly different motion pattern, according to a DICOM RT plan, created in the ${\text{Delta}}^{4\text{PT}}$ software. Leaves make a series of major stops, one at every 10 mm. The nominal gap width is of 2 cm. At each major stop, three measurements are considered at three locations around the diodes. The first one is delivered with the detector row at the center of the 2 cm gap with no offset, the second one with a small offset to the one direction, and the third one with the offset to the opposite direction (Fig. 2). A total of 57 segments in 19 positions is delivered.

**Figure 3:**
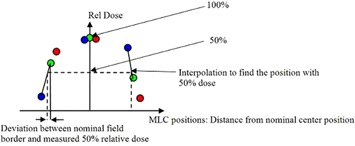
A diagram explaining how the ${\text{Delta}}^{4\text{PT}}$ software calculates the deviation between the nominal and the true leaf position, through the 50% relative dose level around the nominal center position, at each major stop of the leaves.^(15)^

The reason of this motion pattern is that the actual irradiated gap width is estimated through the 50% relative dose level, which defines the radiation field. From the three measurements in each position, an interpolation is performed and the 50% level point is determined. This position is compared with the geometric leaf position and discrepancies are evaluated (Fig. 3).

Although this motion pattern is recommended for the ${\text{Delta}}^{4\text{PT}}$, when applied on a film it does not produce a satisfying image that could be optically examined for discrepancies, due to its complex movements.

## III. RESULTS & DISCUSSION

### A. EBT2 film calibration

**Figure 4:**
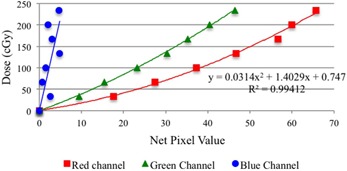
The film calibration curve, showing a greater sensitivity for the red channel. The calibration curve equation for the red channel is showing.

The film calibration curves for the three analyzed color channels are presented in Fig. 4. When the red channel is chosen for further analysis, a greater sensitivity is obtained and a wider range of gray values is covered, in comparison to the blue and green channels. These results are in accordance with previous data published by several authors^11^, ^13^, ^15^ for dose levels up to 10 Gy. Thus, all further analysis of our measurements was made on the red channel.

### B. The Picket Fence test results on the EBT2 film

For the Picket Fence test, the irradiation of the film was performed with 350 MUs, which correspond to a dose on the film high enough to capture a wide range of gray levels (Fig. 4).

**Figure 5:**
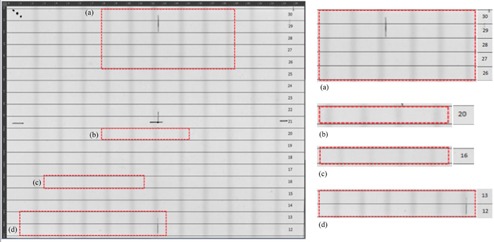
The irradiated EBT2 film and the marked positions where dispositions are magnified. Leaf pairs 12–30 can be seen on the film.

Figure 5 shows the resulting film where the irradiated bands are observed and the visible MLC leaves have been numbered, separated. Leaf pairs 12–30 are visible on the film, since the film dimensions were not adequate to image the whole set of leaves at the isocenter, but covered the same field of view as the ${\text{Delta}}^{4\text{PT}}$ method. Marks were used to indicate the orientation of the film and the center of the field of view (FOV). The regions with discrepancies were identified on the film and magnified. An optical check of the MLCs positioning is possible this way and, with a further analysis, the radiation width and its deviation from the nominal width can be measured.

Detailed optical observation reveals that pair leaves 26–30 show a general trend of an offset to the left and of a wider gap, in comparison to the other leaves. Pair leaves 12 and 13 also show a wider gap. On the contrary, leaf pair 16 presents a narrower irradiated gap and pair leaf 20 shows an offset to the right, in the central irradiated area.

**Figure 6:**
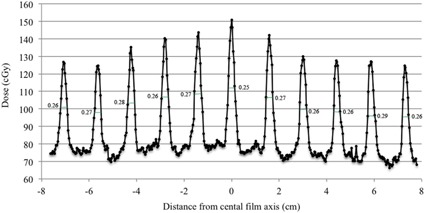
The dose profile, extracted from the gray value profile through the calibration curve equation, of leaf pair 21 throughout its path. The high peaks indicate the dark lines on the film, corresponding to the irradiated bands. The FWHM of each curve (in cm) is measured and compared to the nominal gap width, which is 0.3 cm. Gap deviations of 0.1–0.5 mm are observed.

A further analysis is possible through the dose profile of each pair leaf along its total pathway. The dose profile can easily be extracted from the gray value profile of the film through the calibration curve equation, after background is subtracted. The radiation gap width for each band can be estimated through the full width at half maximum (FWHM) of the narrow peaks which correspond to the irradiated bands. It can then be compared to the nominal gap width, which was chosen to be 0.3 cm. The actual gap width for the central leaf 21, a leaf with no observed disposition with naked eye, is measured as an example. It is found to range between 0.25–0.29 cm, instead of the nominal 0.3 cm. This corresponds to a disposition of 0.1–0.5 mm, which is considered to be within safe limits (Fig. 6).

### C. The Picket Fence test results on the ${\text{Delta}}^{4\text{PT}}$ phantom

**Figure 7:**
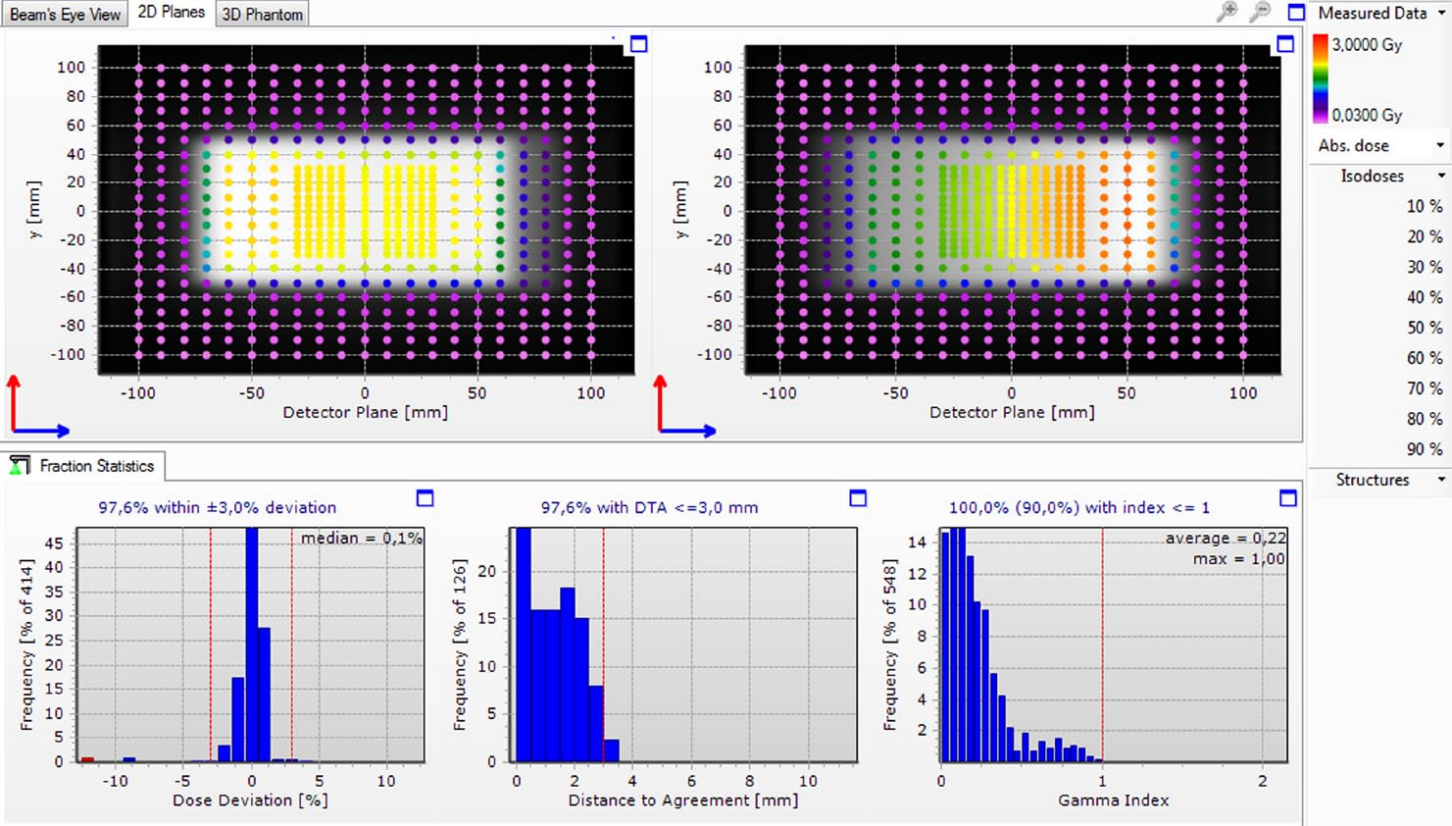
A screen capture of the ${\text{Delta}}^{4\text{PT}}$ software showing the fluence in the two phantom planes after two $10\times 10\;{\text{cm}}^{2}$ isocentric orthogonal irradiations, at 0° and 90° gantry angles, for output and setup correction purposes. Different colors indicated the range of measured dose. The setup correction is based on the measured γ index, determined by the ratio of the dose deviation (DD) and the distance to agreement (DTA) between the planned and the measured dose distribution.

As mentioned above, before each measurement is performed, two irradiations are needed, at 0 and 90 gantry angles, for output and setup correction purposes. The field fluence, as was measured in the output check can be seen in Fig. 7.

**Figure 8:**
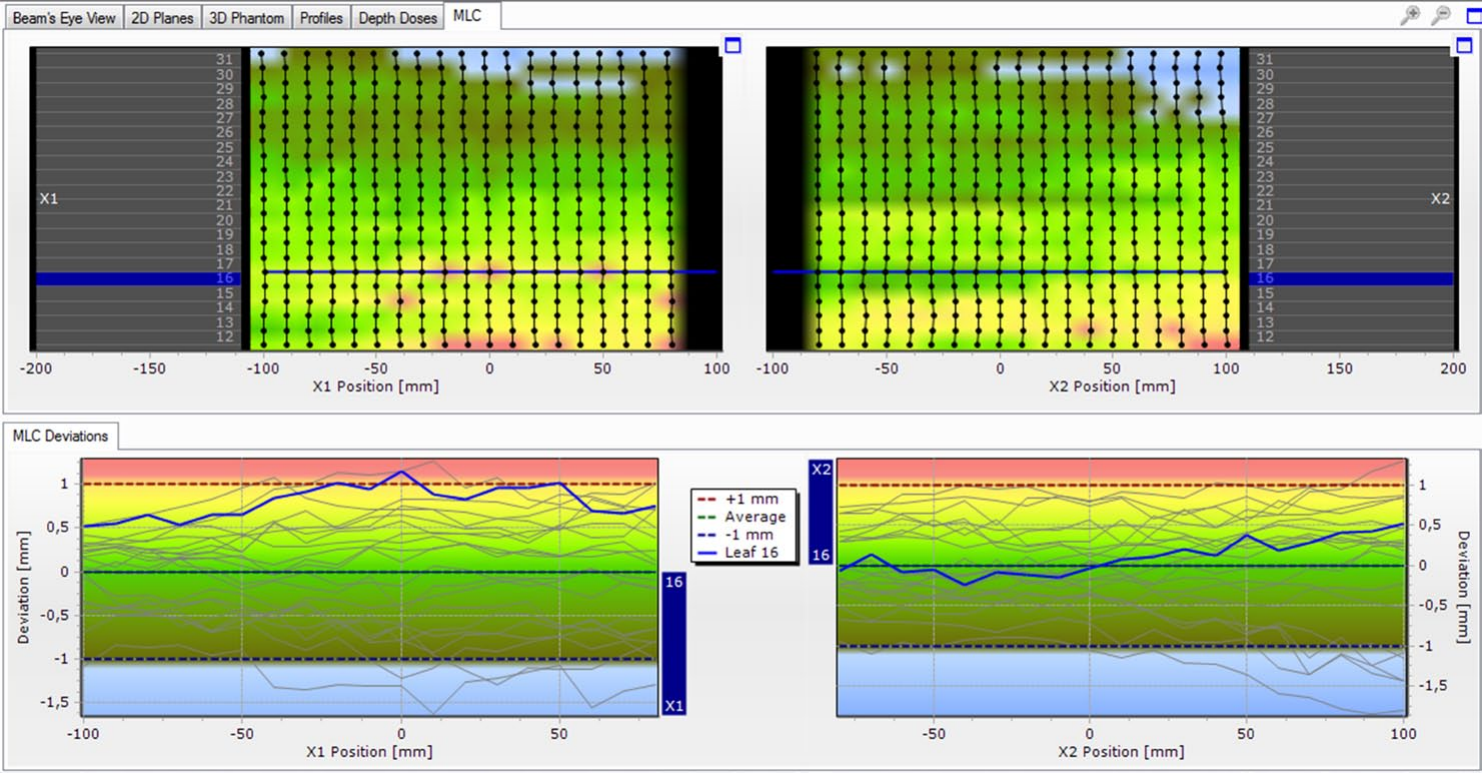
A screen capture of the ${\text{Delta}}^{4\text{PT}}$ software results for the leaf deviations. Positional deviation of each leaf position in comparison to the alignment of the other leaves, for the two leaf banks. Leaf 16 is indicated with the blue line. Dispositions of lower that 1 mm are noted with yellow to dark green shades. Dispositions of greater than 1 mm are noted with either red or blue, depending on their direction (to the left or the right).

Figure 8 shows the results of the ${\text{Delta}}^{4\text{PT}}$ technique. In the top image of Fig. 8, the two MLC leaf banks (right and left set of leaves) and the relative position of each leaf can be seen. In the bottom image each leaf is indicated by a separate line, showing positional deviations throughout its total pathway.

Dispositions of within ±0.5 mm are marked with either dark green (negative disposition to the left) or light green (positive disposition to the right). Dispositions of greater than 0.5 mm are marked with either oily green (negative) or yellow (positive), and those of greater than ±1 mm with either blue (disposition to the left) or red (disposition to the right). Dispositions of even 0.1 mm can be observed with great accuracy.

For leaves 21 and 26–30, an offset to the left is observed. For leaves 12–16, an offset to the right is observed, ranging between 0.5–1 mm.

This is in agreement with the dispositions to the left, observed with the EBT2 film, for leaves 26–30 that can now be determined as significant (greater than 1 mm). The offset of leaves 12–16 was not previously observed, probably because of the relatively small dispositions.

Leaf pairs 12, 13, and 16 also show an offset. These pairs were thought to present only gap width deviations when checked through the film, but it can now be seen that they are also shifted.

Additional slight dispositions (between ±1 mm) can also be observed in other places, not previously observed with the EBT2 film. This is due to the very subtle differences in gray values on the film, produced by so small dispositions, not easily observable with a naked eye.

**Figure 9:**
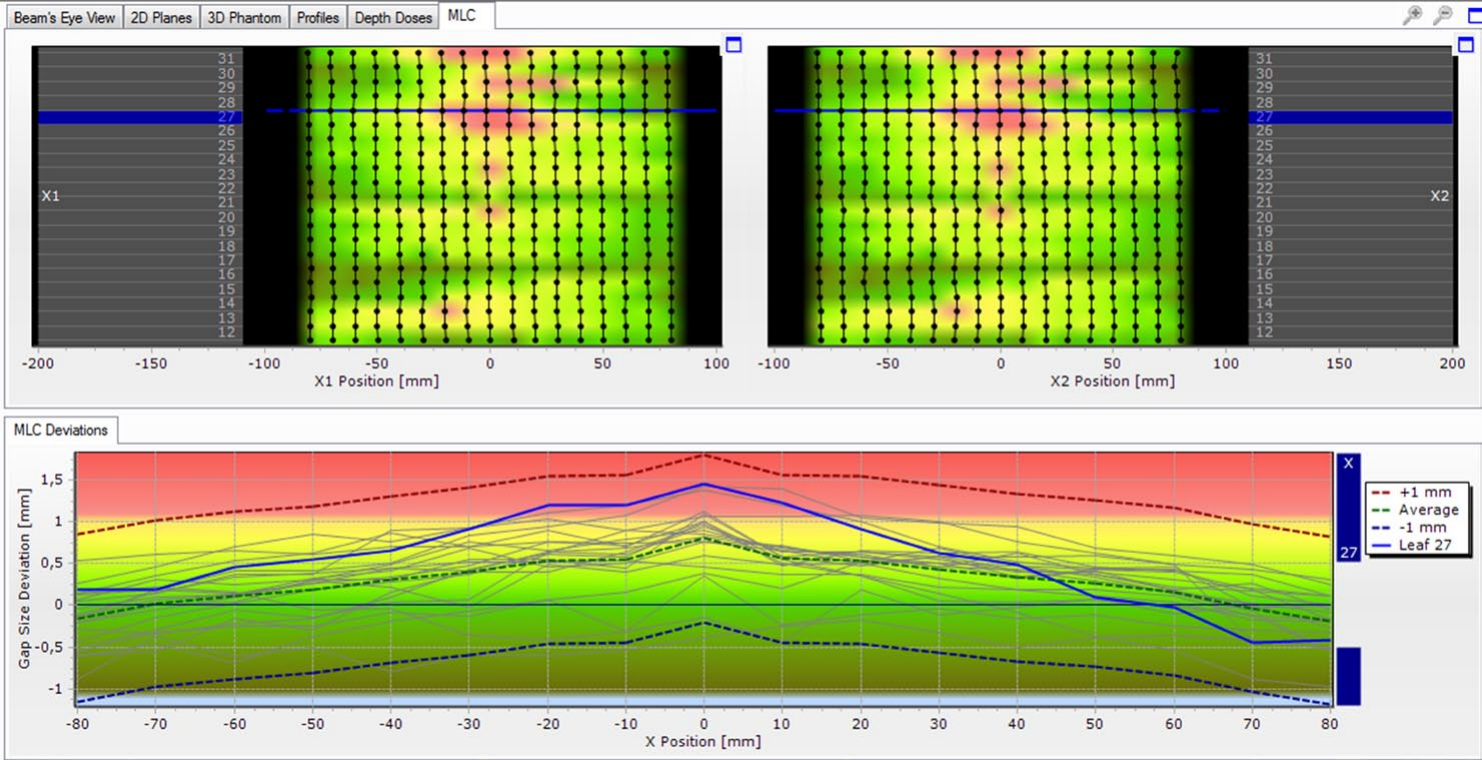
A screen capture of the ${\text{Delta}}^{4\text{PT}}$ software results for the gap deviations. The gap deviation of each leaf pair in comparison to the gaps of the other leaf pairs is shown. Gap deviations of lower that 1 mm are noted with yellow to dark green shades. Deviations of greater than 1 mm are noted with either red or blue, depending on their direction.

Apart from leaf deviations from each band, the combined results of the two banks can also be noted by choosing to examine the resulting gap deviation of each leaf pair. Results are shown in Fig. 9.

The gap deviations previously observed with the film are also confirmed. The wider gap of leaves 26–30 in the central area can now be characterized as significant, and so can the narrower gap of leaf pair 16. Even slighter deviations (<1 mm), as in leaf pairs 12 and 13 are also confirmed.

The gap deviation of the central leaf 21, previously observed through the film analysis, is confirmed and estimated between 0.0−0.5 mm. However, it can be seen that additional slight gap deviations (<1 mm), not previously observed, are now apparent.

## IV. CONCLUSIONS

The two methods are found to be in good agreement and to present important advantages in the clinical routine. The radiochromic film method presents a first order, qualitative QA procedure, whereas the ${\text{Delta}}^{4\text{PT}}$ method a second order quantitative QA process.

When an EBT2 film and the picket fence test are chosen for estimating positional inaccuracies of the MLC leaves, it is found that significant dispositions (greater than 1 mm) can be observed with the naked eye. Some slighter dispositions (0.5–1 mm) can also be observed. A relatively fast qualitative estimation is possible and a further analysis may provide more detailed results. The method though cannot refrain from being observer dependent. The film does not require any additional set up or post processing.

When the ${\text{Delta}}^{4\text{PT}}$ phantom is chosen, even slight dispositions can be accurately and numerically determined. Its software also offers the possibility to check both the gap width and leaf position deviations. A possible limitation of the method arises for new linacs equipped with MLC leaves of width smaller than the 5 mm resolution of the phantom.

In treatment techniques where a high accuracy is required in the delivered dose distribution, such as in IMRT, the use of a device that can produce precise numerical values is recommended for the MLC positional inspection.

The agreement between the results of the two methods, performed at different gantry angles, is also an indication that gravity does not significantly affect the MLC performance. However, possible gravity effects could be further investigated in a more extended study.
